# Prediction model for leaflet thrombosis in patients undergoing transcatheter aortic valve implantation: the EFFORT study

**DOI:** 10.1007/s00392-024-02486-3

**Published:** 2024-07-16

**Authors:** Gloria M. Steiner-Gager, Jovan Rogozarski, Christina Kronberger, Al Medina Dizdarevic, Peter Quehenberger, Ruediger Schernthaner, Christian Loewe, Lukas Reider, Andreas Strassl, Katarina Kovacevic Miljevic, Bernd Jilma, Cihan Ay, Oliver Königsbrügge, Marek Postula, Christian Hengstenberg, Jolanta M. Siller-Matula

**Affiliations:** 1 Department of Medicine II, Division of Cardiology, Medical University of Vienna, Waehringer Guertel 18-20, 1090 Vienna, Austria; 2 Department of Clinical Pharmacology, Medical University of Vienna, Vienna, Austria; 3 Department of Laboratory Medicine, Medical University of Vienna, Vienna, Austria; 4 Department of Department of Biomedical Imaging and Image-Guided Therapy, Medical University of Vienna, Vienna, Austria; 5 Department of Medicine I, Division of Hematology and Hemostaseology, Medical University of Vienna, Vienna, Austria; 6 Department of Experimental and Clinical Pharmacology, Centre for Preclinical Research and Technology (CEPT), Medical University of Warsaw, Warsaw, Poland; 7 Central Radiology Institute, Diagnostic and Interventional Radiology, Klinik Landstraße, Vienna, Austria

**Keywords:** EFFORT score, Predictive score, Leaflet thrombosis, Aortic stenosis, TAVI, TAVR, vWF activity, LDH, Hemoglobin, Oral anticoagulation, MDCT

## Abstract

**Background:**

Leaflet thrombosis (LT) is a multifaceted and underexplored condition that can manifest following transcatheter aortic valve implantation (TAVI). The objective of this study was to formulate a prediction model based on laboratory assessments and clinical parameters, providing additional guidance and insight into this relatively unexplored aspect of post-TAVI complications.

**Methods:**

The present study was an observational prospective hypothesis-generating study, including 101 patients who underwent TAVI and a screening for LT (the primary endpoint) by multidetector computed tomography (MDCT). All images were acquired on a third-generation dual-source CT system. Levels of von Willebrand factor (vWF) activity, hemoglobin (Hb), and lactate dehydrogenase (LDH) were measured among other parameters. A predictive score utilizing binary logistic regression, Kaplan–Meier time-to-event analysis, and receiver operating characteristics (ROC) analysis was established.

**Results:**

LT (11 subclinical and 2 clinical) was detected in 13 of 101 patients (13%) after a median time to screening by MDCT of 105 days (IQR, 98–129 days). Elevated levels of vWF activity (> 188%) pre-TAVI, decreased Hb values (< 11.9 g/dL), as well as increased levels of LDH (> 312 U/L) post-TAVI and absence of oral anticoagulation (OAC) were found in patients with subsequent LT formation as compared to patients without LT. The established EFFORT score ranged from − 1 to 3 points, with an increased probability for LT development in patients with ≥ 2 points (85.7% of LT cases) vs < 2 points (14.3% of LT cases; *p* < 0.001). Achieving an EFFORT score of ≥ 2 points was found to be significantly associated with a 10.8 times higher likelihood of developing an LT (*p* = 0.001). The EFFORT score has an excellent c-statistic (area under the curve (AUC) = 0.89; 95% CI 0.74–1.00; *p* = 0.001) and a high negative predictive value (98%).

**Conclusion:**

An EFFORT score might be a helpful tool to predict LT development and could be used in risk assessment, if validated in confirmatory studies. Therefore, the score has the potential to guide the stratification of individuals for the planning of subsequent MDCT screenings.

**Graphical abstract:**

Central illustration. Created with BioRender.com

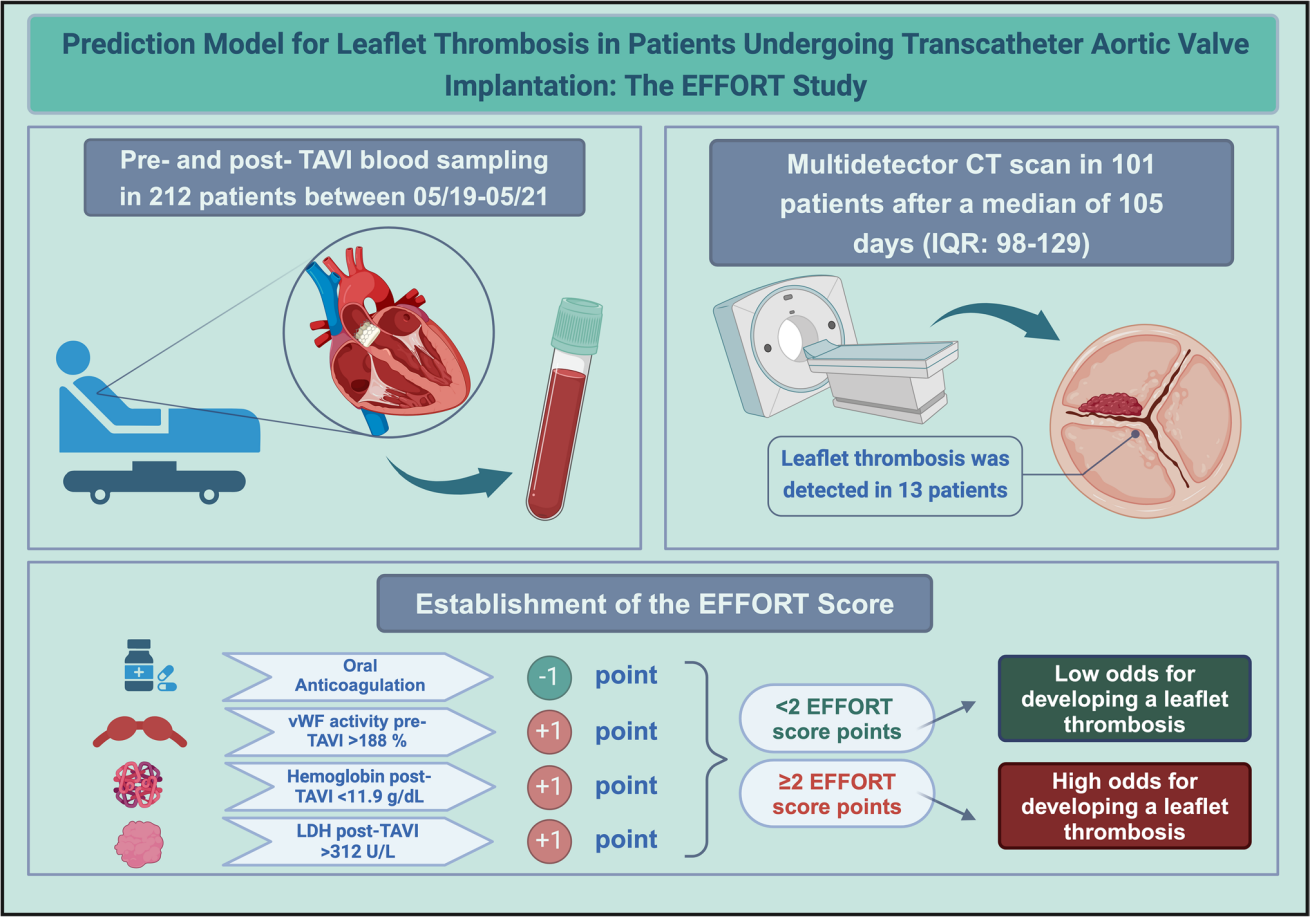

## Introduction

Aortic stenosis (AS) stands as the predominant valvular pathology in economically developed regions, with transcatheter aortic valve implantation (TAVI), alternatively known as transcatheter aortic valve replacement (TAVR), emerging as a pivotal intervention alongside surgical approaches [1–3]. Recent attention has been directed toward the occurrence of leaflet thrombosis (LT) following TAVI [4], a condition that may manifest as either subclinical (SLT), denoting asymptomatic cases, or symptomatic presentations (clinical LT) [5]. Importantly, however, LT is linked to an elevated risk of stroke [6], which further underscores the increased importance of implementing an optimized antithrombotic regimen following TAVI [7]. Currently, patients without indication for oral anticoagulation (OAC) typically receive lifelong therapy with aspirin [8]. Regarding LT diagnosis, multidetector computed tomography (MDCT) has emerged as the preferred diagnostic modality [9]. In this context, MDCT scans may reveal various pathological manifestations. Isolated leaflet thrombosis involving one or more leaflets is termed hypoattenuated leaflet thrombosis (HALT). A more severe manifestation, characterized by diminished leaflet motion, is identified as reduced leaflet mobility/motion (RELM). The combination of HALT and RELM is designated as hypo-attenuation affecting motion (HAM) [6].

Various predictors for LT have been proposed [10]. However, until now, there has been no clear guideline to identify which patients require specific follow-up procedures, such as routine MDCT to optimize patient follow-up care and antithrombotic regimen. Therefore, the aim of the current study was to propose a scoring model based on routine/easily obtained laboratory assessments and clinical parameters to assist clinicians in identifying specific patient populations requiring additional attention due to the potentially higher risk of LT development.

## Methods

### Study design

**Fig. 1:**
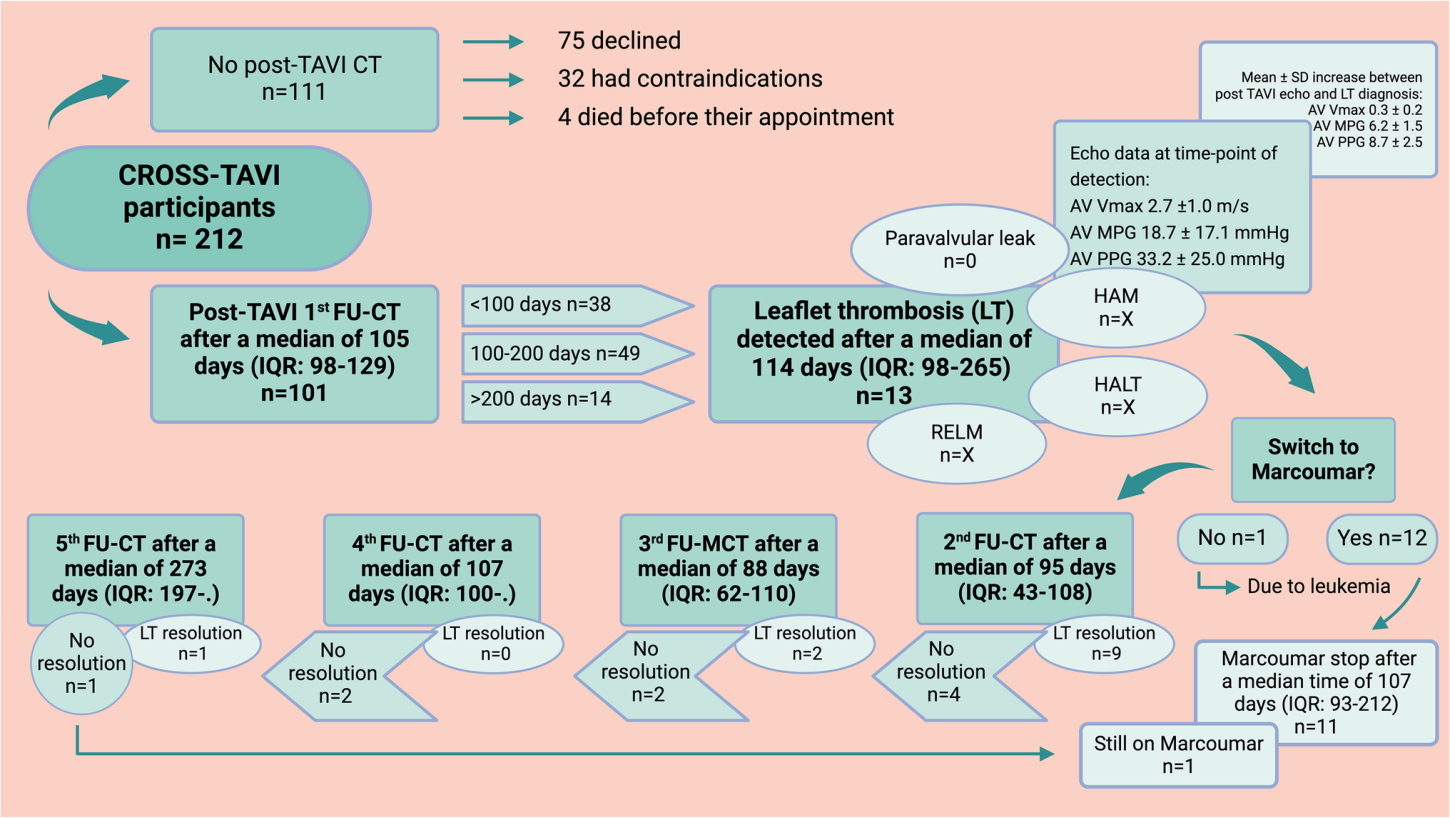
Flow chart of study data and study procedures. TAVI, transcatheter aortic valve implantation; MDCT, multidetector computed tomography; FU, follow-up; LT, leaflet thrombosis; HAM, hypo-attenuation affecting motion; HALT, hypoattenuated leaflet thickening; RELM, reduced leaflet motion; AV Vmax, aortic valve maximum velocity; AV MPG, aortic valve mean pressure gradient; AV PPG, aortic valve peak pressure gradient; SD, standard deviation; IQR, interquartile range; VKA, vitamin K antagonist. Created with BioRender.com

The EFFORT (PrEdictors For LeaFlet ThrOmbosis afteR TAVI) substudy was a prospective, non-randomized, observational investigation conducted within the framework of the ongoing CROSS-TAVI study. The CROSS-TAVI study aims to identify predictors of clinical outcomes and leaflet thrombosis, assess the natural history of thrombosis, and evaluate its association with different therapies. Additionally, correlations between imaging techniques, biomarker levels, genetic factors, and neurological outcomes will be explored. The substudy cohort comprised of consecutive patients diagnosed with severe AS who were scheduled for TAVI and subsequently underwent MDCT for the purpose of detecting LT. The EFFORT substudy was conducted at the Medical University of Vienna from May 2019 to October 2022. The study protocol adheres to the principles outlined in the Declaration of Helsinki and received approval from the Ethics Committee of the Medical University of Vienna (approval number: 1970/2018). The primary objective of the EFFORT substudy was to explore the incidence and predictors of LT (both clinical and subclinical) in individuals undergoing TAVI. Additionally, the study aimed to develop a scoring system to guide the identification of patients for whom a routine MDCT scan would be beneficial. Inclusion criteria comprised of individuals diagnosed with severe AS, scheduled for TAVI with a minimum age of 50 years, and the capability to provide written informed consent. The diagnosis of severe AS adhered to universally accepted criteria [3]. Among the 212 participants of the CROSS-TAVI trial, MDCT was selectively performed in 101 individuals, accounting for 48% of the total cohort. The decision to employ MDCT in specific cases was influenced by various factors, as detailed in Fig. 1. Since the definite diagnosis of (subclinical) LT is made via MDCT scans [11], the EFFORT substudy only included patients, where an MDCT scan was performed.

### MDCT scanning protocol

Images were acquired on a third-generation dual-source CT system (SOMATOM Force, Siemens Healthineers, Forchheim, Germany) with patients in supine position, cranio-caudal scan direction, and an inspiratory breath-hold instruction. For cardiac synchronization, a helical retrospective ECG-gated scan mode was used; images were acquired during a full cardiac cycle. In the course of radiation dose reduction, the scan range was limited to the aortic valve, not covering the whole heart. Automatic tube voltage and tube current selection were turned on (Care kV, CareDose4D, Siemens Healthineers, Forchheim, Germany). For vessel opacification, Visipaque 320 (GE Healthcare, Chicago, IL) was injected, and volumes and flow rates were adapted according to the automatic voltage selection. Correct scan timing was ensured using the bolus-tracking technique and a post-threshold delay of 8 s. Multi-planar reformations and maximum intensity projections were reconstructed; detailed acquisition and reconstruction parameters are provided in Table 1.

**Table 1:** MDCT scanning parameters

Image acquisition	
Collimation (n × mm)	192 × 0.6
Reference voltage (kVp)	100
Reference current (mAs)	288
Rotation time (ms)	250
Pitch factor (HR dependent)	0.2
Image reconstruction	
Slice thickness (mm)	0.6
Reconstruction increment (mm)	0.4
Kernel	Bv40
Matrix size	512 × 512
Iterative reconstruction	ADMIRE level 3

*mm* millimeter, *kVp*, kilovoltage peak, *mAs* milliampere-seconds, *ms* milliseconds, *HR* heart rate

### Study endpoints

The primary endpoint was defined as the diagnosis of LT via MDCT scan, whether subclinical (detected through MDCT during screening) or clinical (evidenced by symptoms and confirmed by MDCT), after approximately 3 months post-TAVI. Secondary endpoints included differences in laboratory assessments and clinical parameters in patients with or without LT. We further aimed to establish a predictive score to stratify the risk of LT formation following TAVI.

### Laboratory assessment

Blood samples were taken during the intrahospital period both preceding and subsequently after TAVI. Routine laboratory analyses were conducted in accordance with the directives of the attending physician. Additionally, blood specimens were collected for inclusion in our biobank analyses. The biobank of the Medical University of Vienna represents a joint venture comprising of collaborating clinical institutes and is one of the largest biobanks in Europe. Its aim is the establishment of well-characterized and quality-defined collections of tissues and viable cells as a sustainable research resource.

Preceding TAVI, the blood collection (into *VACUETTE®* tubes) comprised of 6 mL K3EDTA, 3.5 mL sodium citrate 3.2%, and 8 mL CAT serum separator clot activator. Post-TAVI, a total of three blood collecting tubes were utilized, including one tube of 6 mL K3EDTA, one tube of 3.5 mL sodium citrate 3.2%, and one tube of 8 mL CAT serum separator clot activator. Routine laboratory parameters were analyzed according to the standard operating procedures, established by the Department of Laboratory Medicine at the Medical University of Vienna. Further, von Willebrand factor (vWF) activity, vWF antigen, D-dimer, and prothrombin fragment (F1 + 2) levels before and after TAVI were evaluated by the Department of Laboratory Medicine, using blood samples obtained from the biobank specimens.

For the assessment of vWF activity, citrate plasma was analyzed using the vWF:GPIbM assay. This assay involves the binding of vWF to a gain-of-function mutant GPIb fragment [12], as elucidated in detail by [13]. In the same context, vWF antigen levels were quantified through latex agglutination, with the methodology extensively described in [14]. Prothrombin F1 + 2 was determined via ELISA [15], while D-dimer analysis utilized latex agglutination, as outlined in [16].

In addition, we performed quantification for different biomarkers, using commercially available ELISA kits. We utilized *Invitrogen* (*Thermo Fisher Scientific, Inc.,* MA, USA) Human Fatty Acid Binding Protein (FABP3, cardiac) ELISA kits, *Invitrogen* ST2 (interleukin-33 receptor (IL-33R)) ELISA kits, *Invitrogen* HUMAN IL-33 ELISA kits, and *Invitrogen* Human Neprilysin (MME) ELISA kits. All assays were performed according to the manufacturer’s instructions.

Further, endogenous thrombin potential (ETP) was measured via *Technothrombin* fluorogenic assay (*Technoclone*, Vienna, Austria) at the Department of Internal Medicine I, Division of Hematology and Hemostaseology of the Medical University of Vienna. The assay was conducted in accordance with the instructions provided by the manufacturer and is summarized in [17].

### Statistical analysis

With the assumption of a 5% occurrence of LT in individuals having a negative test result, compared to a 25% incidence of LT in those individuals with a positive test result, our calculations suggest that employing a study cohort of at least 98 patients would provide a statistical power of 80% with a two-sided alpha level of < 0.05.

Continuous data are expressed as median and interquartile range (IQR; range from the 25th to the 75th percentile) or mean ± standard deviation (SD), whereas categoric values are presented as numbers (*n*) and percentages (%), as appropriate. Statistical comparisons between groups were conducted using either the Mann–Whitney *U*-test or the independent sample *t*-test, depending upon the normal distribution of the parameters, which was assessed by the Kolmogorov–Smirnov test. Categorical variables were compared by the *X*^2^-test or Fisher’s exact test. For determination of independent parameters influencing the EFFORT score, univariate binary logistic regression analyses were performed. The predictive capacity of the EFFORT score for subsequent LT post-TAVI was assessed via receiver operating characteristic (ROC) analysis. Time-to-event analyses were executed employing Kaplan–Meier curves and the Mantel-Cox regression test. All statistical analyses were conducted using commercially available software (*IBM SPSS Statistics 29*, Chicago, IL, USA).

## Results

### Baseline characteristics

Table 2 provides a comprehensive overview of the study’s population baseline characteristics. Overall, well-balanced frequencies regarding risk factors, comorbidities, past medical history, concomitant medication, laboratory results, and TAVI-related data were observed between the LT group (*n* = 13, 13%) and the non-LT group (*n* = 88, 87%). Half of the study cohort (50%) consisted of female patients, and the median age was 81 years. The majority of participants presented with various common conditions, including coronary artery disease (71%), arterial hypertension (91%), dyslipidemia (75%), heart failure (75%), and upfront heart failure with preserved ejection fraction (HFpEF; 48%). Diabetes mellitus was less prevalent, occurring in 38% of the total population. A higher proportion of patients without LT suffered from atrial fibrillation (AF) as compared to patients, diagnosed with LT (44% vs 15%; *p* = 0.047). The historical medical backgrounds were comparable between the two cohorts. In terms of medication, aspirin, P2Y12 inhibitors, β-blockers, and spironolacton were administered in about 60% of the study population. Angiotensin-converting enzyme (ACE) inhibitors or angiotensin receptor blockers (ARBs) were the most commonly prescribed antihypertensives with approximately 79%. In contrast, only one patient (1%) was on an angiotensin receptor-neprilysin inhibitor (ARNI). Statins were given to 80% of the study population. Oral anticoagulants (OAC) were more commonly used by patients who did not develop LT as opposed to those with LT (50% vs 15%; *p* = 0.019). The majority of patients were treated with non-vitamin K antagonist oral anticoagulants (NOACs; *p* = 0.010). With respect to laboratory results, most parameters were distributed evenly across patients with or without LT, except for vWF activity pre-TAVI, hemoglobin (Hb) post-TAVI, and lactate dehydrogenase (LDH) post-TAVI. In terms of TAVI-related data, similar results between patients with or without an LT were observed too. The majority of participants (85%) had a classical severe AS. The remaining patients suffered from low-flow, low-gradient AS (14%), and only one patient was diagnosed with paradoxical low-flow, low-gradient AS (1%). Supra-annular and intra-annular leaflet position of the valve did not significantly differ between both groups—similar to echo data before TAVI, valve size, and type or access site for TAVI.

**Table 2:** Baseline characteristics

	Overall, *n* = 101 (100)	Leaflet thrombosis, *n* = 13 (13)	No leaflet thrombosis, *n* = 88 (87)	*p*-value
EFFORT score parameters				
*Oral anticoagulation*	46 (46)	2 (15)	44 (50)	**0.019**
*vWF activity pre-TAVI (%)*	155.5 ± 51.2	198.9 ± 27.3	150.9 ± 51.1	**0.015**
*Hemoglobin post-TAVI (g/dL)*	11.1 ± 1.6	10.2 ± 1.5	11.3 ± 1.6	**0.029**
*LDH post-TAVI (U/L)*	250.2 ± 94.0	331.2 ± 200.8	238.2 ± 59.0	**0.046**
Age (years), mean ± SD	80.7 ± 5.2	79.5 ± 6.5	80.9 ± 5.0	0.369
Sex (female), *n* (%)	50 (50)	8 (62)	42 (48)	0.353
Risk factors/comorbidities, *n* (%)				
Body mass index (kg/m^2^), mean ± SD	30.6 ± 36.7	25.6 ± 5.4	31.4 ± 39.2	0.113
Coronary artery disease	72 (71)	10 (77)	62 (71)	0.630
Peripheral artery disease	17 (17)	3 (23)	14 (16)	0.519
Arterial hypertension	92 (91)	13 (100)	79 (90)	0.227
Dyslipidemia	76 (75)	11 (85)	65 (74)	0.402
Diabetes mellitus	38 (38)	3 (23)	35 (40)	0.246
Atrial fibrillation	41 (41)	2 (15)	39 (44)	**0.047**
Heart failure	75 (75)	10 (77)	65 (75)	0.864
HFrEF	22 (22)	2 (15)	20 (23)	0.537
HFmrEF	5 (5)	0 (0)	5 (6)	0.375
HFpEF	48 (48)	8 (62)	40 (46)	0.295
Smoking	43 (43)	5 (39)	38 (43)	0.748
Family history of CAD	25 (25)	3 (23)	22 (25)	0.881
Past medical history and procedures				
Prior aortic valve intervention	6 (6)	1 (8)	5 (6)	0.775
Prior ACS	11 (11)	3 (23)	8 (9)	0.131
Prior stroke	8 (8)	0 (0)	8 (9)	0.257
Concomitant medication				
Aspirin	61 (60)	10 (77)	51 (58)	0.192
P2Y12 inhibitors	63 (62)	8 (62)	55 (63)	0.947
NOAC	41 (41)	1 (8)	40 (46)	**0.010**
Edoxaban 60 mg, od	11 (11)	1 (8)	10 (11)	0.692
Edoxaban 30 mg, od	7 (7)	0 (0)	7 (8)	0.292
Apixaban 5 mg, bid	10 (10)	0 (0)	10 (11)	0.200
Apixaban 2.5 mg, bid	8 (8)	0 (0)	8 (9)	0.257
Rivaroxaban 20 mg, od	1 (1)	0 (0)	1 (1)	0.699
Rivaroxaban 15 mg, od	1 (1)	0 (0)	1 (1)	0.699
Dabigatran 150 mg, bid	1 (1)	0 (0)	1 (1)	0.699
Dabigatran 110 mg, bid	2 (2)	0 (0)	2 (2)	0.583
VKA	5 (5)	1 (8)	4 (5)	0.625
ß-blockers	58 (58)	8 (62)	50 (57)	0.748
ACE inhibitors/ARBs	80 (79)	10 (77)	70 (80)	0.828
ARNIs	1 (1)	0 (0)	1 (1)	0.699
Calcium channel-blockers	29 (29)	5 (39)	24 (27)	0.405
Loop diuretics	45 (45)	6 (46)	39 (44)	0.901
Spironolacton	61 (60)	10 (77)	51 (58)	0.192
Statins	81 (80)	13 (100)	68 (77)	0.055
Antidiabetic drugs	34 (34)	3 (23)	31 (35)	0.387
Pre-TAVI laboratory data				
White blood cell count (× 10^9^/L)	7.0 ± 1.9	7.5 ± 2.0	6.9 ± 1.9	0.577
Platelets (× 10^9^/L)	214.3 ± 67.5	224.0 ± 71.5	213.0 ± 67.2	0.348
Hemoglobin (g/dL)	13.7 ± 14.5	11.8 ± 1.8	14.0 ± 15.5	0.338
Ferritin (μg/L)	172.4 ± 168.7	200.5 ± 197.3	167.9 ± 164.7	0.813
Transferrin (mg/dL)	248.1 ± 47.3	225.9 ± 37.4	251.7 ± 48.0	0.105
Transferrin saturation (%)	23.1 ± 9.1	24.3 ± 12.0	22.9 ± 8.6	0.930
C-reactive protein (mg/dL)	0.4 ± 0.6	0.3 ± 0.3	0.4 ± 0.7	0.919
Creatinine (mg/dL)	1.0 ± 0.5	1.2 ± 1.0	1.0 ± 0.3	0.644
BUN (mg/dL)	20.2 ± 7.7	20.2 ± 10.3	20.1 ± 7.3	0.481
LDL (mg/dL)	76.7 ± 34.2	68.1 ± 43.3	78.1 ± 32.5	0.330
LDH (U/L)	200.5 ± 52.6	208.6 ± 23.4	199.3 ± 55.5	0.103
ntproBNP (pg/mL)	2067.0 ± 3252.5	1588.4 ± 1496.4	2131.5 ± 3425.1	0.991
HbA1c (%)	5.9 ± 0.8	6.1 ± 1.1	5.9 ± 0.8	0.787
Post-TAVI laboratory data				
White blood cell count (× 10^9^/L)	9.8 ± 5.2	14.1 ± 12.6	9.2 ± 2.3	0.093
Platelets (× 10^9^/L)	165.0 ± 53.1	145.1 ± 58.2	167.8 ± 52.2	0.596
C-reactive protein (mg/dL)	2.5 ± 3.2	2.5 ± 5.0	2.5 ± 2.9	0.133
Ferritin (μg/L)	374.7 ± 735.6	171.6 ± 171.6	419.8 ± 812.5	1.000
Transferrin (mg/dL)	222.7 ± 50.0	157.0 ±	230.0 ± 47.0	0.400
Transferrin saturation (%)	21.4 ± 21.5	20.8 ±	21.5 ± 22.8	0.800
Biomarkers				
PRE D-dimer (µg/mL)	0.9 ± 1.0	0.7 ± 0.2	0.9 ± 1.0	0.453
PRE Prothrombin F 1 + 2 (pmol/L)	283.3 ± 208.8	303.6 ± 130.8	281.2 ± 216.0	0.245
PRE vWF antigen (%)	180.4 ± 66.5	209.1 ± 46.7	177.4 ± 67.8	0.127
POST D-dimer (µg/mL)	2.9 ± 2.9	4.3 ± 6.5	2.7 ± 1.9	0.876
POST Prothrombin F 1 + 2 (pmol/L)	464.1 ± 287.0	438.3 ± 301.8	468.1 ± 287.2	0.646
POST vWF antigen (%)	226.9 ± 73.5	236.0 ± 53.3	225.4 ± 76.4	0.490
POST vWF activity (%)	196.7 ± 44.2	210.6 ± 34.5	194.6 ± 45.4	0.334
TAVI-related data				
Aortic stenosis subtype				
Classical severe	84 (85)	11 (85)	73 (85)	0.980
Low-flow, low-gradient	14 (14)	1 (8)	13 (15)	0.474
Paradoxical low-flow, low-gradient	1 (1)	1 (8)	0 (0)	0.131
Echo data before TAVI				
AV MPG (mmHg)	44.6 ± 13.7	40.1 ± 8.3	45.3 ± 14.3	0.991
AV PPG (mmHg)	71.5 ± 21.9	65.2 ± 16.1	72.4 ± 22.6	0.941
AV area (cm^2^)	0.7 ± 0.2	0.7 ± 0.2	0.7 ± 0.2	0.818
AV Vmax (m/s)	4.2 ± 0.6	4.0 ± 0.5	4.2 ± 0.6	0.929
Valve type				
Self-expandable valve	47 (47)	5 (39)	42 (48)	0.532
Acurate Neo	42 (42)	4 (31)	38 (43)	0.397
Evolut	4 (4)	0 (0)	4 (5)	0.433
Portico	1 (1)	1 (8)	0 (0)	0.129
Baloon-exandable valve	54 (54)	8 (62)	46 (52)	0.532
Sapien 3	30 (30)	4 (31)	26 (30)	0.928
Sapien 3 Ultra	24 (24)	4 (31)	20 (23)	0.525
Leaflet position				
Supra-annular	45 (46)	4 (31)	42 (48)	0.252
Intra-annular	55 (55)	9 (69)	46 (52)	0.252
Valve size				
Small	15 (15)	3 (23)	12 (14)	0.393
Medium	47 (48)	7 (54)	40 (47)	0.622
Large	37 (37)	3 (23)	34 (40)	0.253
Access site				
Transfemoral	101 (100)	13 (100)	88 (100)	1.000
Valve-in-valve TAVI	5 (5)	1 (8)	4 (5)	0.625
Sentinel device	76 (75)	10 (77)	66 (75)	0.881
Bicuspid aortic valve	2 (2)	0 (0)	2 (2)	0.579

Bold *p*-values indicate statistical significance

*vWF* von Willebrand factor, *TAVI* transcatheter aortic valve implantation, *LDH* lactate dehydrogenase, *HFrEF* heart failure with reduced ejection fraction, *HFrmEF* heart failure with mildly reduced ejection fraction, *HFpEF* heart failure with preserved ejection fraction, *CAD* coronary artery disease, *ACS* acute coronary syndrome, *NOAC* non-vitamin K antagonist anticoagulant, *od* once daily, *bid* twice daily, *VKA* vitamin K antagonist, *ACE* angiotensin-converting enzyme, *ARB* angiotensin receptor blocker, *ARNI* angiotensin receptor-neprilysin inhibitor, *BUN* blood urea nitrogen, *LDL* low density lipoprotein, *ntproBNP* N-terminal prohormone of brain natriuretic peptide, *HbA1c* hemoglobin A1C, *prothrombin F 1* + *2* prothrombin fragment 1 + 2, *AV MPG* aortic valve mean pressure gradient, *AV PPG* aortic valve peak pressure gradient, *AV Vmax* aortic valve maximum velocity

### Follow-up procedures and data

Post-TAVI procedures and related data are summarized in Fig. 1. In brief, 101 patients received a follow-up MDCT scan after a median period of 105 days (IQR, 98–129 days) following TAVI. Mean heart rate at MDCT was 66 beats per minute (bpm; range 34–123 bpm). For the majority of patients (*n* = 60), the tube voltage was reduced to 70 or 80 kV by the scanner. This facilitated optimal contrast with only 40 mL of contrast media injected at a flow rate of 3.2 mL/s. The remaining patients were scanned with a tube voltage of 90 or 100 kV, requiring 60 mL of contrast media injected at a flow rate of 4.0 mL/s. Median tube current was 465 mAs (IQR 302–540 mAs). The median dose-length product was 464 mGy × cm (IQR 288–764 mGy × cm), resulting in a median effective dose of 6.5 mSv (IQR 4.0–10.7 mSv).

**Fig. 2:**
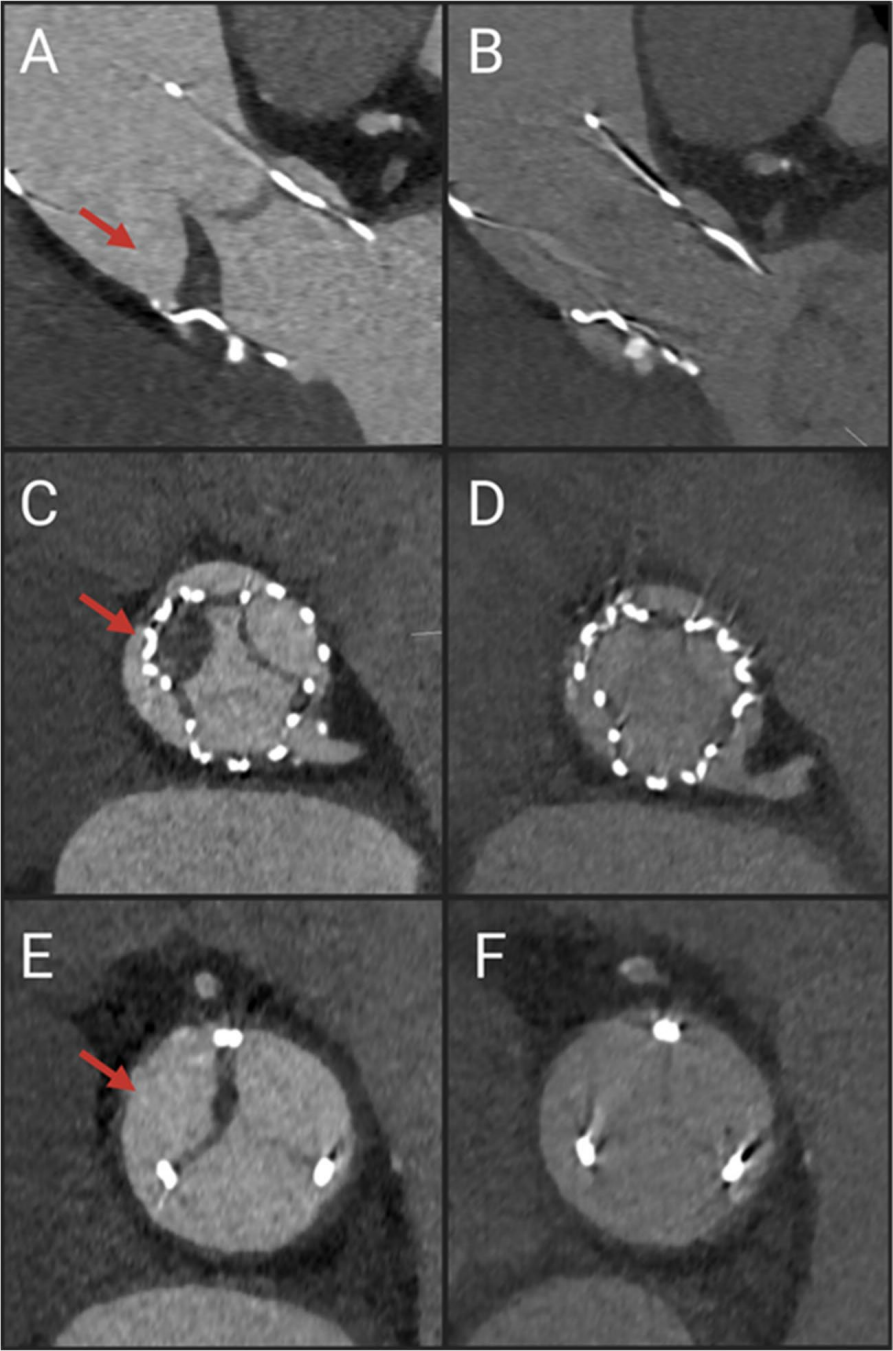
**A**, **C**, **E** Pre- and **B**, **D**, **F** post-resolution imaging of leaflet thrombosis following vitamin K antagonist administration. Coronal and axial plane imaging comparison (**A**, **B** coronal plane; **C**–**F** axial plane). Red Arrows represent leaflet thrombosis

Out of these 101 patients, 13 patients (13%) were diagnosed with an LT, among them, 2 (15%) presented with a clinical LT characterized by severe AS at the time of diagnosis. In contrast, 11 LT patients (85%) showed no symptoms and were diagnosed during screening. None of the patients exhibited a paravalvular leak. However, HAM and HALT were observed in 7 (54%) and 13 (100%) patients, respectively. Conversely, RELM exhibited a graduated pattern with 5 patients (38%) manifesting a mild form, 3 patients (23%) demonstrating moderate expression of RELM, and 3 (23%) experiencing a severe form. Notably, in one case (8%), leaflets were entirely immobile. Consistently, patients with LT exhibited a mean increase of 6.2 mmHg in mean pressure gradient (MPG), 8.7 mmHg in peak pressure gradient (PPG), and 0.3 m/s in peak aortic jet velocity (AV Vmax) between the post-TAVI echocardiogram and echo at the time-point of LT diagnosis via MDCT scan. Following the detection of LT, VKA was prescribed for nearly all patients (92%), with the exception of one participant, who suffered from leukemia and subsequent thrombocytopenia. Median time of VKA administration comprised of 107 days (IQR, 93–212 days). After the second follow-up MDCT scan, LT was resolved in 9 patients (69%). Four patients required a third follow-up MDCT scan after a period of 3 months, revealing LT resolution in 2 (50%) patients. Only in 2 remaining participants a fourth and fifth follow-up MDCT scan was performed, wherein LT resolution was observed in one individual. Figure 2A, C, and E visually represents LT prior to intervention with VKAs, while Fig. 2B, D, and F illustrates the subsequent LT resolution. The comparison is presented in both the coronal plane (depicted by images A and B) and the axial plane (depicted by images C–F). As of the present day, LT remains unresolved in one patient, necessitating an ongoing VKA therapy for this subject. Significantly, this patient underwent a valve-in-valve procedure, and the diagnosis was established over a year later (373 days) due to a clinical LT. Adherence to OAC was assessed during all follow-up visits.

### Distribution of predictive parameters across patients with or without leaflet thrombosis

**Fig. 3:**
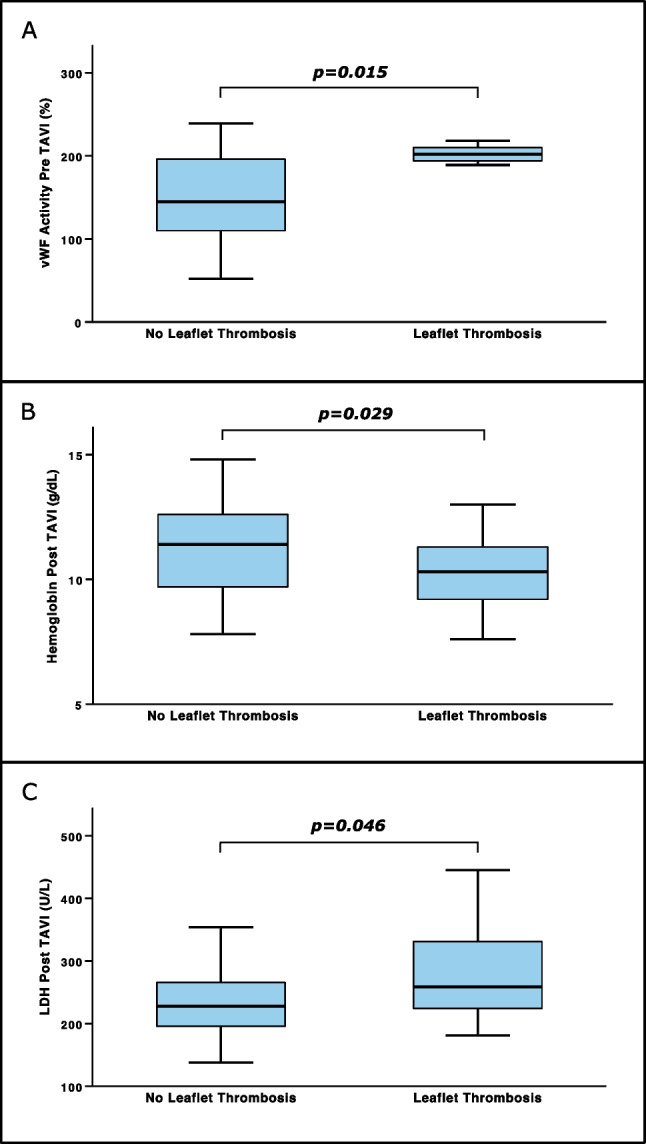
**A** Levels of von Willebrand factor (vWF) activity pre transcatheter aortic valve implantation (TAVI), **B** hemoglobin levels post-TAVI, and **C** lactate dehydrogenase (LDH) values post-TAVI in regard to status of leaflet thrombosis

As described previously and depicted by Fig. 3a, vWF activity pre-TAVI was distributed heterogeneously across patients who were diagnosed with LT as opposed to patients who were not. A median vWF activity level of 202% with an IQR between 189 and 218% was measured in patients with LT. In contrast, patients without LT had about 29% lower values of vWF activity prior to TAVI (median, 144.5%; IQR, 109.5–196.3%; *p* = 0.015). On the other hand, patients with LT showed a roughly 10% reduction in Hb levels post-TAVI (median, 10.3 g/dL; IQR, 9.1–11.4 g/dL) as compared to patients without LT (median, 11.4 g/dL; IQR, 9.7–12.6 g/dL; *p* = 0.029; Fig. 3b). In turn, Fig. 3c displays that patients, who suffered from LT, had an approximately 12% increased LDH level (median, 259 U/L; IQR, 218.5–378.5 U/L) post-TAVI in contrast to those patients where no LT was detected (median, 228 U/L; IQR, 196–266.3 U/L; *p* = 0.046).

### Cut-off stratification of EFFORT score parameters

Based on the ROC analysis, cut-off values for the score parameters were calculated. Hereby, the greatest sum of sensitivity and specificity of the ROC coordinate points was determined. Accordingly, the cut-off value for vWF activity before TAVI was established at 188%. Hemoglobin and LDH post-TAVI showed cut-off points of 11.9 g/dL and 312 U/L, respectively.

### Establishment of the EFFORT score

Depending on the cut-off points of the heterogeneously distributed parameters in patients with and without LT and presence or absence of OAC, a risk score was generated. The score incorporated vWF activity pre-TAVI, Hb as well as LDH post-TAVI and treatment with OAC and evaluated their predictive ability for LT in patients following TAVI. In detail, vWF activity > 188%, Hb < 11.9 g/dL, and LDH > 312 U/L were allocated one point each if the corresponding criteria were met. Contrarily, if treatment with OAC was present, one point was deducted. Consequently, the EFFORT score ranged from − 1 to 3 points, indicating a significantly higher risk of developing LT in individuals with a score of 2 or more points as compared to a score of less than 2 points. Overall, in line with the univariate binary logistic regression, the odds for having an LT after TAVI were 73.2 times higher (95% CI, 7.30–733.76; *p* < 0.001) with an EFFORT score of ≥ 2 points as compared to less than 2 points (Table 3).

**Table 3:** Univariate binary logistic regression analyses for establishing the EFFORT score (excerpt of the data)

Variable	Regression coefficient	OR	95% CI		*p*-value
			Lower	Upper	
*Sex*	0.56	1.75	0.53	5.78	0.357
*Age (continuous)*	− 0.05	0.95	0.85	1.06	0.366
*Body mass index (continuous)*	− 0.09	0.92	0.80	1.05	0.208
*Leaflet position (intra- vs supraannular)*	0.72	2.05	0.59	7.17	0.259
*Valve size (S, M, L)*	− 0.53	0.59	0.26	1.37	0.218
*Aortic stenosis subtype (classical severe, LFLG, paradoxical LFLG)*	0.44	1.56	0.43	5.71	0.502
*Valve-in-valve*	0.56	1.75	0.18	16.99	0.629
*Absence of oral anticoagulation*	**1.71**	**5.50**	**1.15**	**26.27**	**0.033**
*ST2 pre-TAVI (%, continuous)*	− 0.00	1.00	0.99	1.00	0.244
*FABP3 pre-TAVI (%, continuous)*	0.00	1.00	1.00	1.00	0.159
*Neprilysin pre-TAVI (%, continuous)*	0.00	1.00	0.99	1.01	0.633
*vWF Antigen pre-TAVI (%, continuous)*	0.01	1.01	1.00	1.02	0.235
*vWF Antigen post-TAVI (%,continuous)*	0.00	1.00	0.99	1.01	0.687
*Prothrombin F1* + *2 pre-TAVI (pmol/L, continuous)*	0.00	1.00	1.00	1.00	0.786
*Prothrombin F1* + *2 post-TAVI (pmol/L, continuous)*	0.00	1.00	1.00	1.00	0.771
*D-Dimer pre-TAVI (µg/mL; continuous)*	− 0.32	0.72	0.21	2.51	0.611
*D-Dimer post-TAVI (µg/mL; continuous)*	0.13	1.14	0.94	1.37	0.190
*vWF Activity pre-TAVI* > *188%*	**2.85**	**17.29**	**1.94**	**154.17**	**0.011**
*vWF Activity post-TAVI (%;continuous)*	0.01	1.01	0.99	1.03	0.319
*Hb pre-TAVI (g/dL; continuous)*	− 0.18	0.84	0.60	1.17	0.290
*Hb post-TAVI* < *11.9 g/dL*	**2.12**	**8.31**	**1.03**	**66.75**	**0.046**
*LDH pre-TAVI (U/L; continuous)*	0.00	1.00	0.99	1.01	0.568
*LDH post-TAVI* > *312 U/L*	**1.70**	**5.49**	**1.48**	**20.39**	**0.011**
*EFFORT score* ≥ *2 points*	**4.29**	**73.20**	**7.30**	**733.76**	** < 0.001**

Bold *p*-values indicate statistical significance

*OR* odds ratio, *95% CI* 95% confidence interval, *LFLG* low-flow low-gradient, *TAVI* transcatheter aortic valve implantation, *Hb* hemoglobin, *LDH* lactate dehydrogenase

Furthermore, Table 3 illustrates the application of the binary logistic regression analysis, incorporating diverse laboratory parameters such as vWF activity post-TAVI, Hb pre-TAVI, LDH pre-TAVI, vWF antigen, D-dimer, prothrombin F1 + 2, FABP3, neprilysin, IL-33, and ST2 values both pre- and post-TAVI. Notably, the results of the ETP analyses did not attain statistical significance (data not shown).

In addition, the analysis also integrated clinical parameters, including sex, age, body mass index, comorbidities, concomitant medication, and TAVI-related data, such as AS subtype, valve type/size, and leaflet position. Nevertheless, statistical significance was not observed for most parameters, with the exception of treatment with OAC, emerging as a predictor for the development of LT.

### Distribution of EFFORT score points in regard to leaflet thrombosis status

**Fig. 4:**
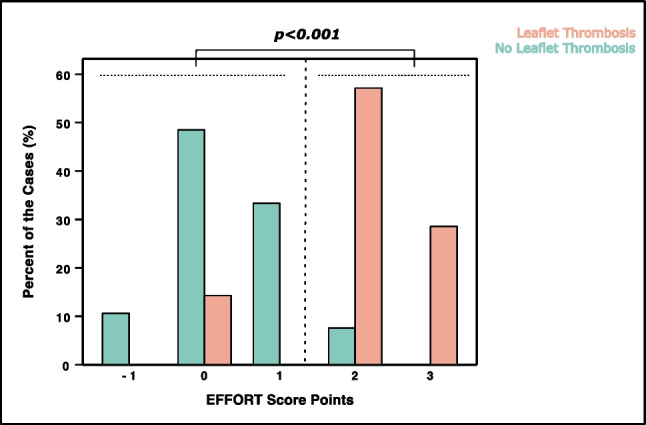
Distribution of EFFORT score points and corresponding incidence of leaflet thrombosis in transcatheter aortic valve implantation (TAVI) patients

Figure 4 demonstrates the significant higher percentage of LT in patients with an EFFORT score ≥ 2 as compared to an EFFORT score of less than 2 points (85.7% vs 14.3%; *p* < 0.001). In detail, 10.6% of the patients without an LT diagnosis had a score of − 1 points; in contrast, not a single patient with an LT had − 1 points. Among patients without LT, the majority (48.5%) had a score of 0, whereas only one patient (14.3%) with LT scored 0 points. Similarly, 33.3% of LT patients obtained a score of 1, whereas none with LT reached 1 point. In turn, solely 7.6% had an EFFORT score of 2 and no LT as opposed to 57.1% of patients with an LT and a score of 2 points. Correspondingly, 28.6% of the LT patients presented with 3 points, contrary to 0% without LT and 3 points.

### Time to event analysis of the EFFORT score

**Fig. 5:**
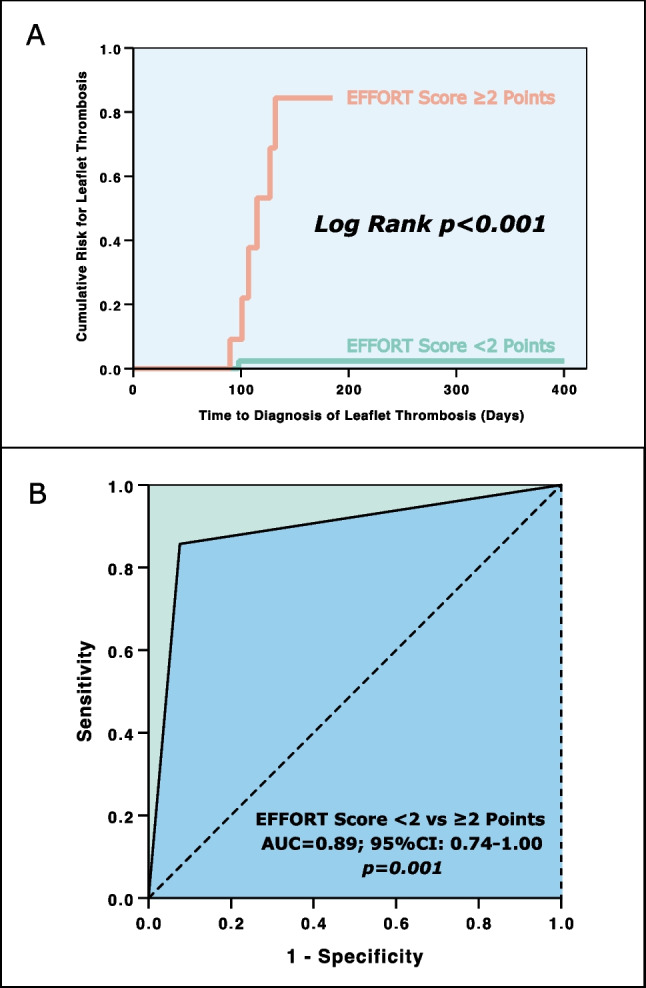
**A** Kaplan–Meier analysis: time-to-event assessment of leaflet thrombosis incidence in EFFORT score groups (< 2 vs ≥ 2 points) and **B** receiver operating curve (ROC) analysis for the EFFORT score to predict leaflet thrombosis after transcatheter aortic valve implantation (TAVI)

Assessment of the EFFORT score was performed in 73 patients out of the entire study population (*n* = 101, 72%) due to missing data of the remaining 28 participants. Among these 73 patients, 62 subjects (85%) achieved an EFFORT score of less than 2 points, while 11 participants (15%) scored 2 or more points (Table 4). The primary clinical endpoint of LT was detected in 7 patients (10%) within the analyzed subgroup (*n* = 73). Only one of these patients (2%) exhibited an EFFORT score of less than 2 points; however, 6 patients (55%) had a score of 2 or more points. Thus, according to the time-to-event analysis, patients with an EFFORT score of ≥ 2 points were 27.5 times more likely to develop an LT as compared to those patients with < 2 points during the median time to screening period of 105 days (log-rank *p* < 0.001; Fig. 5a).

**Table 4:** Event data

Event	EFFORT score assessment, *n* = 73 (100)	EFFORT score < 2 points, *n* = 62 (85)	EFFORT score ≥ 2 points, *n* = 11 (15)		*p*-value
*Leaflet thrombosis*	7 (10)	1 (2)		6 (55)	** < 0.001**

Bold *p*-values indicate statistical significance, *n*, ( %)

### Predictive capability of the EFFORT score for leaflet thrombosis

As shown by Fig. 5b, the EFFORT score exhibited an excellent [18] area under the curve (AUC; c-index) of 0.89 (95% CI, 0.74–1.00; *p* = 0.001) for the prediction of LT following TAVI. Further, the sensitivity and specificity reached 86% and 92%, respectively. The EFFORT score obtained an outstanding negative predictive value of 98% and a positive predictive value of 55%. Positive likelihood ratio and negative likelihood ratio were calculated with 10.8 and 0.2, respectively (Table 5).

**Table 5:** Statistical estimates for leaflet thrombosis prediction based on the EFFORT score

	Leaflet thrombosis *n* = 7 (10%)								
	Test								
	c-Index (95% CI)	*p*-value	Cut-off value	Sensitivity, %	Specificity, %	Positive predictive value, %	Negative predictive value, %	LR +	LR-
*EFFORT score* < *2 vs* ≥ *2 points*	0.89 (0.74–1.00)	**0.001**	2	86	92	55	98	10.8	0.2

Bold *p*-values indicate statistical significance

*95% CI* 95% confidence interval, *LR* + positive likelihood ratio, *LR − *negative likelihood ratio

## Discussion

The principal findings of this study, centered on blood-derived biomarkers and clinical parameters to formulate a potential prediction score for LT in patients post-TAVI, can be succinctly summarized as follows:

i) Preexisting treatment with OAC had protective effects against LT formation.
ii) vWF activity pre-TAVI was elevated in patients who subsequently developed LT.
iii) Hb levels after TAVI were diminished in patients who subsequently developed LT.
iv) LDH value post-TAVI was increased in patients who subsequently developed LT.
v) An EFFORT score of ≥ 2 points was associated with 73 times higher odds of developing an LT than an EFFORT score of less than 2 points.

To the best of our knowledge, this study represents the first attempt to establish a prediction score for LT by integrating laboratory findings and clinical parameters in patients undergoing TAVI. While several studies have explored potential predictors for LT after TAVI, the overall available data remain limited. Some studies have investigated TAVI-related LT predictors, such as balloon-expandable prostheses [19], valve-in-valve procedures [19], supra-annular implantation [20], or larger prostheses [21, 22]. Other factors, associated with an increased risk, include obesity [23], history of smoking [24], or male sex [25]. However, the insignificance of factors, such as valve size or valve type in our study, may be attributed to the smaller patient cohort and is in contrast to the larger studies [19, 22]. In contrast, AF was associated with a decreased risk of LT [25], likely attributed to the inherent anticoagulant effect of preexisting anticoagulation, which itself is linked to a reduced risk of LT [23]. Our findings further support this observation, as preexisting OAC treatment was connected to a lower likelihood of LT formation in our study. Similarly, the GALILEO-4D trial [26], a substudy of the GALILEO trial [27], demonstrated that treatment with rivaroxaban was associated with lower incidence of subclinical leaflet abnormalities as compared to an antiplatelet treatment regimen (2.1% vs 10.9%; *p* = 0.01). Noteworthy, OAC is serving as the preferred therapeutic approach in patients suffering from LT [6]. Overall, none of these parameters, except for AF and OAC, reached statistical significance in our analyses, which might be due to the relatively smaller patient cohort in our study.

Although several laboratory parameters, such as NT-proBNP, prothrombin activation fragment 1 + 2, D-dimer, or platelet extracellular vesicles, have been proposed as potential indicators of an increased risk for LT formation [10], to date, no scoring system incorporating routine laboratory biomarkers and clinical parameters for LT prediction is available. This deficiency presents challenges, particularly due to the lack of standardized approaches in screening for (subclinical) LT [28]. Additionally, the question remains regarding the appropriateness of routine MDCT scans post-TAVI and specifically identifying those patient cohorts, where such scans are justified, and those where they are unnecessary. In the following subsections, we will delve into the parameters of the EFFORT score, providing an in-depth analysis of our findings and exploring potential explanations.

### Increased vWF activity prior to TAVI in patients with LT

In our current investigation, elevated vWF activity prior to TAVI was linked to an increased likelihood of developing an LT. The association between an enhanced vWF activity and increased risk of thrombus formation is already well-established [29, 30]. Even within the realm of TAVI, the significance of vWF and variations in its activity before and after the procedure have been described. Studies have consistently reported higher levels of vWF antigen and activity post-TAVI as compared to pre-TAVI, a trend in line with our own findings [31, 32]. One plausible explanation for these observations is the consideration of vWF as an acute phase reactant [33]. Another possibility is that the increase in vWF activity after TAVI might be attributed to the resolution of Heyde’s syndrome, an acquired vWF deficiency resembling type 2A von Willebrand disease [34, 35]. Approximately 3% of individuals diagnosed with AS also experience this syndrome, characterized by gastrointestinal bleeding caused by angiodysplasia. Pathophysiologically, the high shear stress caused by the AS leads to an increased proteolytic cleavage of high-molecular-weight vWF multimers by ADAMTS13. This results in an impaired hemostasis due to the depletion of functional vWF multimers, consequently elevating the risk of bleeding [31, 32, 36–38]. A pivotal diagnostic parameter to test for von Willebrand disease type 2A is the vWF activity, which is typically decreased in this particular subtype [39]. Our findings align with this pattern, as patients exhibited reduced vWF activity levels before TAVI. Noteworthy, a subsequent increase in vWF antigen and activity post-TAVI has been reported [31, 34, 40], a trend also supported by our results.

Nevertheless, in our study, only the vWF activity before TAVI proved to be a reliable predictor of LT. The post-interventional vWF activity, on the other hand, may be significantly affected by the aforementioned factors, rendering it an unreliable predictor. In our assays, the normal range for vWF activity falls between 48 and 170%. Consequently, patients, who later experienced LT in our study, exhibited vWF levels above the normal range even before TAVI (198.9% ± 27.3%), which were higher than those observed in patients who did not develop an LT (150.9% ± 51.1%). Several potential explanations for the elevated pre-TAVI levels of vWF antigen and activity in patients with subsequent LT are existing, including associations with hypertension, peripheral vascular disease, dyslipidemia, coronary heart disease, or prior ACS [41]. Although not statistically significant due to the balanced baseline characteristics, a tendency toward an increased occurrence of these conditions is observed in patients who subsequently develop LT as compared to those who do not in our study cohort. However, further research regarding this issue is warranted.

Overall, this leads to the assumption that patients with elevated vWF activity preceding TAVI are prone to develop a subsequent LT, and routine testing of vWF activity prior to TAVI might be employed for risk stratification.

### Decreased Hb following TAVI in patients with LT

The present study has further unveiled an association between lower levels of post-TAVI Hb and an increased risk of subsequent LT formation. Anemia and risk for thrombosis have been brought into context previously. One study found an increased susceptibility to symptomatic venous thromboembolism in acutely ill patients with low Hb values [42]. Another study identified anemia as a risk factor for cerebral venous thrombosis [43]. Additionally, some reports proposed an association between anemia and arterial thromboses [44, 45]. However, comprehensive data concerning this issue are scarce. One study described how iron-deficiency anemia might enhance thrombotic tendency through mechanisms, such as thrombocytosis and sustained platelet activation [46]. Interestingly, our study observed a lower mean platelet count in patients after TAVI (165 ± 53 × 10^9^/L) compared to pre-TAVI (214 ± 68 × 10^9^/L), with an even more pronounced reduction in patients after TAVI who subsequently developed LT (145 ± 58 × 10^9^/L) in contrast to those who did not (168 ± 52 × 10^9^/L). This platelet reduction shortly after TAVI might be caused by reactions related to the contrast agent [47]. Significantly, there were no discernible variations in ferritin, transferrin, and transferrin saturation between patients who experienced LT and those who did not. However, a separate study demonstrated comparable outcomes between patients with iron-deficiency anemia and those with non-iron-deficiency anemia in patients after TAVI [48].

An alternative hypothesis that may account for our findings is the potential role of anemia itself in promoting a prothrombotic state through its impact on blood flow. Reduced Hb levels and the resulting decrease in oxygen-carrying properties can result in inadequate tissue oxygenation, a factor of particular significance during situations of heightened metabolic demands. As a consequence, blood flow velocity increases to counteract for the oxygen deficiency associated with anemia, which in turn may lead to hemodynamic alterations, including turbulence, which might itself contribute to thrombus formation [49–51]. An additional consideration for the decreased hemoglobin levels could be attributed to hemolysis following TAVI, which is prevalent in 9–28% depending on the type of valve employed, the definition of hemolysis, and the literature [52, 53].

### Increased LDH following TAVI in patients with LT

Another parameter contributing to the EFFORT score was the observation of increased LDH levels following TAVI in patients subsequently experiencing LT. While the association between LDH and an elevated risk of thrombosis is not firmly established in the context of TAVI, a study published in the *New England Journal of Medicine* highlighted elevated LDH levels in patients, developing pump thrombosis following left ventricular assist device (LVAD) implantation. Incidents of pump thrombosis exhibited a peak around the first month post-implantation and declined by the sixth month [54]. This temporal pattern aligns with LT occurrences after TAVI, diagnosed in about 30% of cases 3 to 4 months post-procedure [6]. Further, they emphasized a rapid increase in LDH levels, occurring within the initial weeks following LVAD implantation, frequently anticipated and served as an early indicator of pump thrombosis [54], which is also supported by another investigation [55]. Notably, LDH has evolved into a routine biomarker employed for monitoring individuals with LVADs [56]. The pathophysiological mechanisms underlying LDH and thrombus formation are complex and comprehensively reviewed by Gordon et al. [57]. In summary, thrombosis and hemolysis are intertwined processes. According to the Virchow Triad, certain conditions, including changes in blood flow, alterations in blood composition, and surface properties where blood comes into contact, can contribute to clot formation. Particularly, LVADs impair normal blood flow, potentially triggering the formation of blood clots, which can subsequently result in hemolysis [57]. Conversely, free Hb generated by hemolysis exerts inhibitory effects on ADAMTS13, leading to a reduced degradation of vWF, ultimately promoting a prothrombotic state [58]. This, on the other hand, could also account for the observed tendency toward elevated levels of vWF antigen/activity and the decreased Hb values after TAVI in patients who later developed LT as opposed to those who did not, as described before. The sequential relationship between thrombosis and hemolysis, however, and the causative factors are not easily determined [58].

To our knowledge, there are currently no studies documenting the relationship between LDH and thrombosis following TAVI. Nevertheless, the possibility that LDH serves as an LT predictor may underly a similar mechanism, especially when considering TAVI as an exogenous element within cardiovascular system, similar to LVAD.

Last but not least, the question arises whether isolated SLT (HALT) warrants treatment with systemic OAC, considering evidence suggesting that HALT is not inherently associated with elevated mortality risk, as it may spontaneously resolve [59, 60]. However, data regarding this issue is conflicting, as another study has indeed shown an association between HALT and long-term mortality [61]. Furthermore, our meta-analysis, with over 11,000 patients, unequivocally demonstrated a 2.6-fold increased risk for stroke (RR, 2.56; 95% CI, 1.60–4.09; *p* < 0.00001) in this population, underscoring the therapeutic benefit of OAC in managing SLT [6].

In summary, we want to emphasize the novelty of the EFFORT score in predicting LT in patients after TAVI, utilizing routine biomarker assessments and clinical parameters. The EFFORT score exhibits an outstanding negative predictive value of 98%, signifying a remarkably low likelihood of LT development following TAVI when the score is less than 2 points. If the results of our study are confirmed, patients with a score of 2 or more points might undergo a screening MDCT for LT. This approach can be potentially associated with lower costs and limits unnecessary radiation and the use of contrast media. Our study advocates for the validation of the scoring system to assess its applicability in decision-making processes for identifying patients who would derive benefits from a screening MDCT for LT after TAVI. Integrating the EFFORT score into the conventional post-TAVI regimen could offer a viable strategy to optimize patient treatment, as OAC represents the therapy of choice for LT. However, routine OAC administration after TAVI was shown to be associated with worse outcome [27], underscoring the importance of LT detection to guide optimal medical therapy. Further, future validation of the EFFORT score in an independent study cohort could provide additional guidance and insight into this relatively unexplored aspect of post-TAVI complications.

## Limitations

Our investigation identifies several limitations. Firstly, there is a potential for bias inherent in the observational study design, although all baseline characteristics are distributed homogeneously across both subgroups, except for atrial fibrillation. In addition, our analyses incorporated patients who underwent valve-in-valve procedures; although distributed evenly across both subgroups and reflecting real-life data, this could introduce indeterminate bias. Additionally, our analysis did not strictly adhere to the VARC-3 (Valve Academic Research Consortium 3) criteria in its reporting [62]. Secondly, the number of events applicable for the application of the EFFORT score is limited to 10%, which may pose challenges in constructing a robust predictive model. Nevertheless, this proportion aligns closely with findings from a large meta-analysis, reflecting an overall incidence of LT of 8% (95% CI, 5–13%) [63]. Further, the essential step of validating our findings in an independent study cohort, crucial for ensuring the broader applicability of the results, has yet to be undertaken. Not least, there was a significant proportion of patients in the CROSS-TAVI study who did not undergo CT, despite justifiable reasons; however, this could still lead to selection bias.

## Conclusion

In this hypothesis-generating study, we established the EFFORT score as a predictive tool for the occurrence of LT following TAVI. Incorporating the EFFORT score into standard post-TAVI treatment might represent a feasible approach to assess and identify patients who should undergo a routine MDCT scan within 3 to 4 months following the procedure.

## Data Availability

Data will be made available on request.

## Abbreviations

TAVI: Transcatheter aortic valve implantation
LT: Leaflet thrombosis
MDCT: Multidetector computed tomography
LDH: Lactate dehydrogenase
Hb: Hemoglobin
vWF: Von Willebrand factor
OAC: Oral anticoagulation
HALT: Hypoattenuated leaflet thrombosis
HAM: Hypo-attenuation affecting motion
RELM: Reduced leaflet mobility/motion

## Declarations

### Impact on daily practice

LT formation in patients after TAVI is associated with an increased risk for stroke.

Integrating the EFFORT score into the conventional post-TAVI regimen could offer a viable strategy to identify patients who would benefit from a MDCT scan within 3 to 4 months post-procedure. Future validation of the EFFORT score in an independent study cohort could provide additional guidance and insight into this relatively unexplored aspect of post-TAVI complications.
